# Macrophages Polarized by Expression of ToxoGRA15_II_ Inhibit Growth of Hepatic Carcinoma

**DOI:** 10.3389/fimmu.2017.00137

**Published:** 2017-02-13

**Authors:** Yuanling Li, Faustina Poppoe, Jian Chen, Li Yu, Fang Deng, Qingli Luo, Yuanhong Xu, Yihong Cai, Jilong Shen

**Affiliations:** ^1^Department of Pathogen Biology and Provincial Laboratories of Pathogen Biology and Zoonoses, Anhui Medical University, Hefei, China; ^2^Department of Microbiology and Immunology, School of Medical Sciences, University of Cape Coast, Cape Coast, Ghana; ^3^Department of Laboratory Medicine, Provincial West Hospital, Anhui Medical University, Hefei, China; ^4^Diagnostic Laboratory of the First Affiliated Hospital, Anhui Medical University, Hefei, China; ^5^Clinical Laboratory, Anhui Medical University, Hefei, China

**Keywords:** *Toxoplasma gondii*, dense granule protein GRA15, hepatocellular carcinoma, tumor-associated macrophages, polarization

## Abstract

A growing body of evidence suggests that tumor-associated macrophages are deeply involved in the hepatocellular carcinoma proliferation and account for the large proportion of infiltrated cells in tumor tissues and play a major role in promotion of tumor growth. On the other hand, studies have demonstrated that *Toxoplasma gondii* virulence-associated molecule of dense granule protein (ToxoGRA15_II_) tends to induce classically activated macrophages (M1) differentiation. Thus, we explored the M1 induced by ToxoGRA15_II_ *in vitro* and its inhibitory impact on the proliferation, invasion, and metastasis of hepatic carcinoma in murine model. Here, we constructed recombinant plasmid of *pegfp-gra15_II_* and subsequently ligate it to lentivirus (Lv) vector, with which RAW264.7 was transfected. The results showed that the transfected macrophages were polarized to M1. Coculture of the M1 with Hepa1-6 cells showed a remarkable inhibition of migration and invasion of the tumor cells and decreased expressions of matrix metalloproteinase (MMP)-9 and MMP-2 without notable apoptosis of Hepa1-6 cells. Subsequently, ToxoGRA15_II_-polarized macrophages inoculated to tumor-bearing C57BL/6 mice were seen in both spleen and tumor tissues, and tumor growth was sharply restricted. Particularly, interleukin-6 (IL-6) expression, which is closely associated with the cancer malignant behaviors, was significantly dampened in tumor tissues. In addition, expression of TNF-α and IL-12 mRNAs was increased, whereas IL-6 and interleukin-10 mRNAs were downregulated in splenocytes. Our results indicate that the effector molecule of ToxoGRA15_II_ may induce macrophage polarization to M1 that has a restrictive effect on tumor growth *via* its related cytokines profile in tumor and spleen tissues. Besides, ToxoGRA15_II_, due to its early activation of specified cell population and non-toxicity to mammalians, has a potential value for a novel therapeutic strategy of enhancing host innate immunity against tumor development.

## Introduction

Hepatocellular carcinoma (HCC) ranks in the top five diagnosed malignant cancers in men and in the seven in women (1, 2). In the last decades, the mortality of liver cancer has not been noticeably reduced although phenomenal advances have been made in the technologies of diagnosis and treatments, such as surgery, radiation, and chemotherapy. Recently, immunotherapy is considered to be a crucial part of cancer treatments (3). It has been known that the cross talk between the tumor cells and their surrounding microenvironment is pivotal for HCC development. In the tumor microenvironment, tumor-associated macrophages (TAMs) infiltrate into tumor stroma and facilitate tumor proliferation, survival, and migration (4). Plasticity is a hallmark of macrophages. It has been confirmed that macrophages have two different polarized states, one is the classically activated macrophages phenotype (M1) and the other is the alternatively activated macrophages phenotype (M2). The M1 induced by lipopolysaccharides (LPS) and Th1 cytokine interferon-γ (IFN-γ) possess high antigen-presenting ability and secrete inducible nitric oxide synthase (iNOS), which lead to a high level nitric oxide (NO) killing of intracellular pathogens. In contrast, the M2 responding to Th2 cytokine interleukin-4, interleukin-10 (IL-10), and interleukin-13 activation are capable of suppressing immune response (5–7). Affected by tumor microenvironment, TAMs can also be divided into M1 phenotype, which plays a role in antitumorigenic response, and M2 phenotype, which promotes tumorigenesis (6, 8, 9). Thus, TAMs are believed to be the key target in the immunotherapy.

The ability of various infections to inhibit tumor growth by modulating the host immune system has been previously proved. For example, malaria parasite infection, remarkably suppresses Lewis lung cancer growth through innate and adaptive antitumor response (10). Mice inoculated with both *Toxoplasma* and cancer cells presented significantly increased survival rates, CD8+ T cell proportion, IFN-γ mRNA expression levels, serum IgG2a titers, and inhibited angiogenesis when compared to the animals with only tumor cells plantation (11).

*Toxoplasma gondii* is an obligatory intracellular parasite that affects any warm-blood animals including humans. Several investigations indicate that approximately one-third of the world’s population is affected by *T. gondii*. The sexual life cycle of *T. gondii* is restricted to the intestinal epithelium of feline (12, 13). Due to the immune surveillance of host, most immunocompetent people infected with the parasite are asymptomatic. The parasite may exist in the cyst form and in the tissues of human body. In immunocompromised individuals, however, such as patients with AIDS and those who have received long-term immunosuppressive treatments, latent *T. gondii* infection may lead to severe consequences of toxoplasmosis, mainly encephalitis (14).

Recent studies have showed that *T. gondii* strains have a rich genetic diversity in geographical regions around the world. Type I (RH, GT1), type II (PRU, ME49), and type III (CTG) are widely distributed in Europe and North America (15, 16). Though the three genotypes of *Toxoplasma* have been identified in the isolates from humans, the majority of human cases are associated with type II strains (17). These strains differ widely in virulence, persistence, and migratory capacity in mice (16). Besides its complex cellular structure, *T. gondii* has three main secretory organelles termed as microneme, rhoptry, and dense granule. These organelles are able to secrete polymorphic effector molecules into the host cytosol to modulate host signaling pathways and link to strain virulence. Macrophages infected with type II strain of *Toxoplasma* are classically activated. This is due to its dense granule protein GRA15_II_, which activates nuclear factor (NF)-κB, drives macrophage to M1 polarization, induces high expression of IL-12, stimulates NK and T cells secreting IFN-γ, and evokes Th1 type immune response (18–20). Interestingly, the virulence-associated effector of ToxoGRA15_II_ may induce M1 phenotype polarization and alleviate fibrogenesis caused by schistosomiasis (21).

In the present study, we observed the mouse macrophage RAW264.7 cell line infected with lentiviral vectors containing *gra15_II_*, which was amplified from the *T. gondii* PRU strain. We found that the RAW264.7 cells were driven toward M1 polarization. GRA15_II_-induced M1 were cocultured with the murine HCC heap1-6 cell line in transwell to clarify the *in vitro* effect of the skewed macrophage phenotype on proliferation, migration, invasion, and the expression changes of matrix metalloproteinases (MMPs). Moreover, the C57BL/6 mice were treated with activated M1 cells *via* high-pressure injection of the tail vein following hepa1-6 cells subcutaneous inoculation. The tumor volume, histopathology, TAMs, immunosuppressive factors, and angiogenesis-related factors were detected, respectively.

## Materials and Methods

### Reagents

The following reagents were used in the study: Dulbecco’s modified Eagle’s medium (DMEM) and fetal bovine serum (FBS) were obtained from Wisent (Montreal, QC, Canada). Puromycin (PM), penicillin, and streptomycin were purchased from Sigma (St. Louis, MO, USA). SDS-polyacrylamide gel electrophoresis and 10% buffered neutral formaldehyde were purchased from Beyotime (Shanghai, China). Nitrocellulose membranes were provided by Millipore (Billerica, MA, USA). FITC-labeled anti-mouse F4/80, PE-conjugated anti-mouse programmed death ligand 1 (PD-L1), and PE-Cy5-labeled anti-mouse CD80 monoclonal antibodies were obtained from eBioscience (San Diego CA, USA) for flow cytometry analysis. Antibodies against MMP-2, MMP-9, and GAPDH used for Western blotting were manufactured by Proteintech (Chicago, IL, USA). Rat anti-mouse F4/80, CD68, IL-10, TGF-β, VEGF, and anti-mouse major histocompatibility complex class II (MHCII) monoclonal antibodies that recognize cells from both BALB/c and C57 BL/6 mice were manufactured by Abcam (Cambridge, MA, USA) for immunohistochemistry assays. The TNF-α enzyme-linked immunosorbent assay (ELISA) kit was provided by CUSABIO (Wuhan, China). The interleukin-6 (IL-6) ELISA kit was obtained from RayBiotech (Norcross GA, USA). The Greiss Reagent System determined for nitrite was purchased from Promega Biotech Company (Madison, WI, USA).

### Parasites, Plasmid Construction, and Lentvirus Infection

*Toxoplasma gondii* tachyzoites of PRU (type II) strain were initially obtained by mouse passages with brain homogenate containing cysts. The open reading frame encoding T*g*GRA15_II_ (omitted signal peptide of 1,500 bp, http://ToxoDB.org) was amplified through real-time (RT)-PCR from the total tachyzoites RNA, recombinant plasmids pEGFP-*gra15_II_* were constructed, and recombinant lentivirus (Lv) vectors (LV-pEGFP-*gra15_II_*) were obtained (21).

### Cell Culture

The murine HCC cells, Hepa1-6, were purchased from the Chinese Academy of Sciences Cell Bank in Shanghai. The murine Mφ cell line, RAW264.7, was preserved in the laboratory. RAW264.7 cells transfected by LV-pEGFP-*gra15_II_* were named LV-*gra15*_II_-Mφ. The mock Lv-infected RAW264.7 cells were termed LV-Mφ. The two cell lines remain high in transfection efficiency when screened with PM. All of these cells were cultured in DMEM supplemented with 10–15% FBS and 1% penicillin–streptomycin at 37°C with 5% CO_2_.

### Flow Cytometry Assay

The transfection and M1 surface marker analysis of macrophages that express GRA15_II_ were determined by flow cytometry. Briefly, single cell suspensions of the three groups of Mφ (LV-*gra15*_II_-Mφ, LV-Mφ, and Mφ) were washed in PBS containing 1% FBS and adjusted to 1 × 10^6^ cells per 100 µl PBS with 1% FBS, respectively. The cells were subjected to FITC-labeled anti-mouse F4/80, followed by PE-conjugated anti-mouse PD-L1, and PE-Cy5-labeled anti-mouse CD80 for surface antigens staining. All cells were incubated with the antibodies at 4°C for 20 min in order to protect the cell from light and washed in PBS twice. Cells were analyzed with flow cytometry. Results were analyzed using FlowJo software.

### MTS Assay

The culture supernatants of LV-*gra15_II_*-Mφ, LV-Mφ, and Mφ, were applied to Hepa1-6 cell *in vitro*, and the Hepa1-6 viability was determined by MTS assay (22). MTS assay CellTiter 96 Aqueous One Solution reagents were manufactured by Promega (Madison, WI, USA). Briefly, LV-*gra15*_II_-Mφ, LV-Mφ, and Mφ cells (2 × 106) were separately cultured for 48 h, and then the supernatants were collected. Hepa1-6 cells were seeded in 96-well plates at a density of 1 × 104 cells per well in 100 μl DMEM supplemented with 10% FBS for 12 h and then exposed to different supernatants for 48 h. At 4 h before culture termination, 20 μl of MTS reagent was added to the wells for 1 h incubation. The absorbance density was read on a 96-well plate reader (BioTek) at wavelength 490 nm.

### Transwell Assay

Transwell devices were used with a 0.4-μm-pore polycarbonate filter membrane, which allow small and soluble molecules but not cells to pass through. Hepa1-6 cells (1.5 × 10^6^) were seeded in lower chamber. LV-*gra15*_II_-Mφ, LV-Mφ, and Mφ (2 × 10^6^) were seeded in the 1% penicillin–streptomycin. After 72 h incubation, the Hepa1-6 cells were harvested for total RNA and protein detections.

### Wound Healing Assay

The migration of Hepa1-6 cells, cocultured with the three groups of macrophages as described above, were assessed using the wound healing assay. Hepa1-6 cells (1 × 10^6^) were inoculated in the lower chambers in the 6-well plate, and the macrophages (2 × 10^6^) were inoculated in the upper chambers at the same time. The upper and the lower chambers were separated for culturing. After 24 h of the cell culture with Hepa1-6 cells nearly at 80% confluence, a 10-µl pipette tip was used to make a straight scratch to simulate a wound, and then washed twice with PBS. At least three randomly selected fields along the scraped line in each well were photographed. Finally, the medium in both upper and lower chambers was changed with fresh DMEM containing 3% FBS. The upper chamber was inserted into the lower chamber for coculture without cellular contact. Following 12 and 24 h incubation, the selected fields were photographed again. The gap distance was quantitatively evaluated using Image-Pro Plus 6.0.

### Invasion Assay

The invasion capability of Hepa1-6 cells after coculturing with various transfected Mφ was assessed using transwell assay. Also, 8-μm-pore polycarbonate filter membrane was used within side of the transwell apparatus. The upper chambers were pre-covered with Matrigel diluted with culture medium and incubated at 37°C overnight according to the manufacturer’s instructions. Then, Hepa1-6 (1 × 10^4^) cells were suspended in 100 μl serum-free DMEM and deposited into the upper chamber per well. The lower chamber contained various types of Mφ (1 × 10^6^) suspended in 700 µl 10% FBS-supplemented medium. After incubation for 24 h, the cells inside of the upper chamber were removed using cotton swabs. The cells on the surface of the polycarbonate filter membrane were fixed in 10% buffered neutral formaldehyde for 15 min and washed twice in PBS. Then, the cells were stained with crystal violet for 15 min after drying at the room temperature. Before the observation, the cells were washed twice in PBS and dried again. The invasion ability of cells was quantified by counting the number of cells that have invaded the membranes in five randomly selected fields under a microscope.

### Animal Care and Ethics Statement

Female C57BL/6 mice (6 weeks old) were purchased from Changzhou Gavens Laboratory Animal Company, China (production permit No. Scxk2013-003). All animal care and experimental protocols were conducted in strict accordance with the Chinese National Institute of Health Guide for the Care and Use of Laboratory Animals (1998) and approved by the Institutional Review Board of Anhui Medical University Institute of Biomedicine (permit No. AMU26-080610). Every effort was made to minimize animal suffering during the study.

### Tumor Models

For the subcutaneous tumor model, 100 µl normal saline containing 3 × 10^6^ Hepa1-6 cells were subcutaneously injected into the right lower inguinal of one naïve mouse. After 3 days, a hard mass could be palpated, and the tumor volume reached 50 mm^3^. Tumors were seen in all inoculated animals. The mice were randomly divided into three groups (10 mice each) as follows: LV-*gra15*_II_-Mφ, LV-Mφ, and normal saline (NS) control. On the third and fifth day of post Hepa1-6 inoculation, the mice of LV-*gra15*_II_-Mφ and LV-Mφ groups received 100 µl NS within 2.5 × 10^6^ corresponding macrophages through tail vein injection, respectively, and the control mice were given the same volume of NS. On days 3, 5, 7, and 10 following Hepa1-6 inoculation, tumor size was measured using vernier caliper and calculated using the formula 0.52 × *a* × *b*^2^ (*a*, long diameter of the tumor; *b*, short diameter of the tumor). Ten days later, the animals were scarified under euthanasia for further analyses.

### RNA Extraction and qRT-PCR

Total RNA from the cocultured cells, tumors, and spleens were extracted using Trizol reagent and reversely transcribed to cDNA using Prime Script first Stand cDNA Synthesis Kit according to the manufacturer’s instructions. The qRT-PCR was performed to examine the expression of TNF-α, IL-12, iNOS, MMP-9, MMP-2, IL-10, IL-6, TGF-β, and VEGF using SYBR Premix Ex Taq kit by the ABI Prism 7500 sequence detection system according to the manufacturer’s guidance. GAPDH gene was used as a reference. All the reactions were run in technical triplicates. The relative mRNA expression was calculated with the comparative ΔCt method using the formula 2^−ΔΔCt^. The forward and reverse primers are listed in Table 1.

**Table 1:** **The primers used for qRT-PCR**.

Target	Forward primer (5′–3′)	Reverse primer (5′–3′)
GAR15	CGCTCGAGAATAATTCGGTGGCTTG	AGGGATCCTTCATGGAGTTACCGCTGATTG
TNF-α	ACGGCATGGATCTCAAAGAC	GTGGGTGAGGAGCACGTAGT
IL-12p40	GATGTCACCTGCCCAACTG	TGGTTTGATGATGTCCCTGA
iNOS	CACCTTGGAGTTCACCCAGT	ACCACTCGTACTTGGGATGC
MMP-2	GAATGCCATCCCTGATAACCT	GCTTCCAAACTTCACGCTCTT
MMP-9	CCTACTGCGGGCTCTTCT	CCTGTAATGGGCTTCCTCT
IL-6	CCGGAGAGGAGACTTCACAG	CATTTCCACGATTTCCCAGA
IL-10	GCTCCTAGAGCTGCGGACT	TGTTGTCCAGCTGGTCCTTT
TGF-β	CTGGATACCAACTACTGCTTCAG	TTGGTTGTAGAGGGCAAGGACCT
VEGF	CAGGCTGCTGTAACGATGAA	AATGCTTTCTCCGCTCTGAA
GAPDH	CAACTTTGGCATTGTGGAAGG	ACACTTTGGGGGTAGGAACAC

*iNOS, inducible nitric oxide synthase; MMP, matrix metalloproteinase; IL-6, interleukin-6; IL-10, interleukin-10*.

### ELISA and Nitrite Assays

LV-*gra15*_II_-Mφ, LV-Mφ, and Mφ were separately seeded in 6-well plates (2 × 10^6^ cells per well), suspended in 2 ml common culture medium, and cultured at 37°C with 5% CO_2_ for 48 h. Cell supernatants were collected and analyzed for cytokine values (TNF-α) and NO concentration. IL-6 in the tumor homogenate supernatants was also determined by ELISA in accordance with the manufacturer’s instructions. The NO content reflected as the nitrite concentration was analyzed using the Griess Reagent System.

### Western Blotting Analysis

Total proteins were extracted from the Hepa1-6 cocultured with the macrophages and separated on 12% SDS-polyacrylamide gels. The proteins were then transferred onto nitrocellulose membranes. Non-specific binding was blocked with 5% skim milk in PBS-Tween-20 (0.1%) for 6 h at room temperature. The membranes were then incubated with primary antibodies to MMP-9 (1:1,000), MMP-2 (1:1,000), and GAPDH (1:2,000) at 4°C overnight and with the horseradish peroxide-conjugated secondary antibodies at room temperature for 2 h. ECL kit was used to detect the specific signals, and the bands intensity was visualized.

### Immunohistochemical Analysis

The tumor tissues from each animal were removed immediately under anesthesia and placed in 10% buffered neutral formaldehyde. First, the antigen retrieval was executed under high pressure in 10mM EDTA buffer. Second, the samples were incubated with primary antibodies to F4/80, CD68, IL-10, TGF-β, VEGF, or MHCII at 4°C overnight. Finally, the sample was incubated for 45 min with secondary antibodies. Bright-field images were photographed and analyzed. The positive macrophages were defined by examining F4/80 and CD68 and were counted in 10 randomly selected fields at ×400 magnification.

### Statistical Analysis

All experiments were replicated three times with similar results. All data are expressed as mean ± SD and evaluated using a two-tailed unpaired Student’s *t*-test. Statistical significance was determined by using GraphPad Prism Software, and *P* < 0.05 was taken as significant.

## Results

### RAW264.7 Cell Line Was Stably Transfected

**Figure 1:**
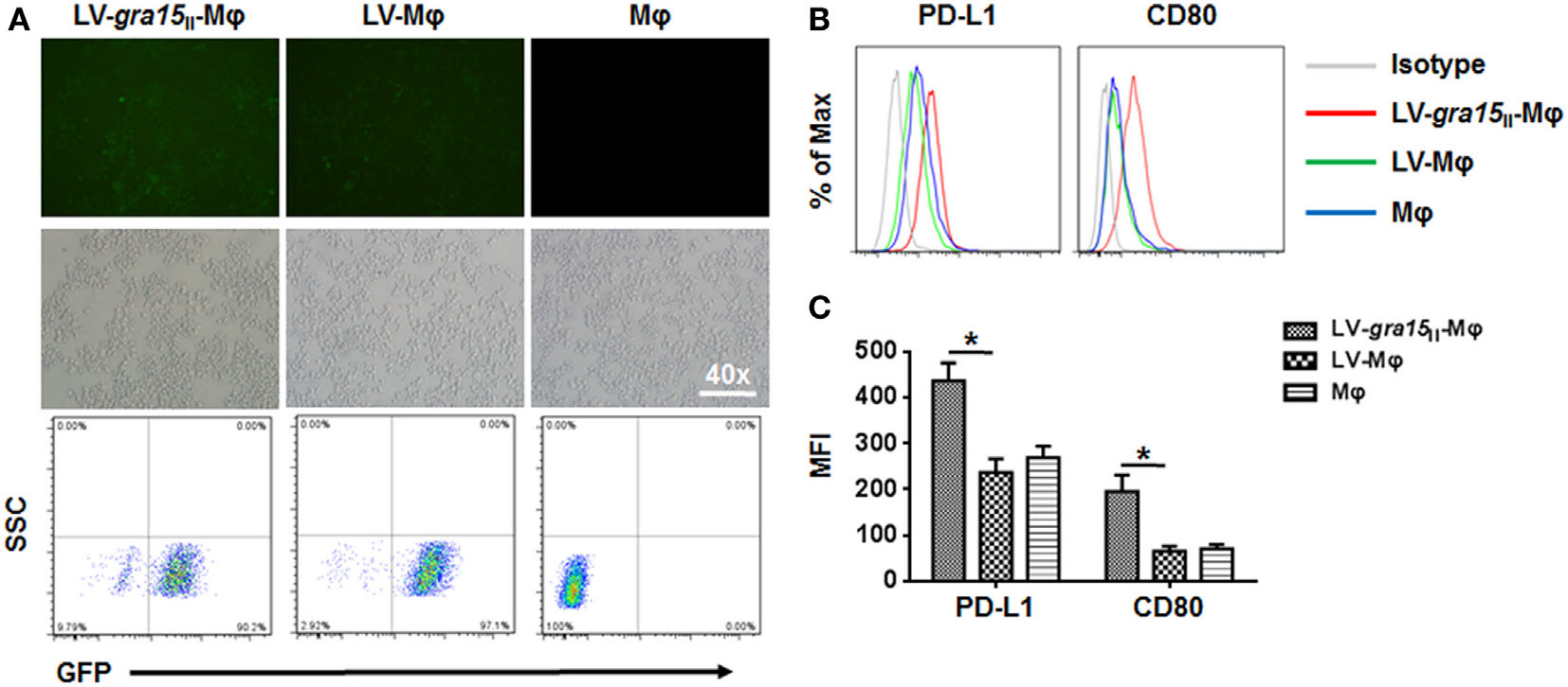
**RAW264.7 cells were infected with GRA15_II_ fused to pEGFP**. **(A)** Green fluorescent protein can be observed by using the fluorescence microscope. Transfection efficiency was measured by flow cytometry. **(B)** LV-*gra15*_II_-Mφ cells surface mark, programmed death ligand 1 (PD-L1) and CD80 determined by flow cytometry. The expressions of PD-L1 and CD80 in LV-*gra15*_II_-Mφ were increased compared with LV-Mφ. **(C)** Statistical analysis of mean fluorescence intensity (MFI) of markers shown in **(B)** (*P < 0.05).

RAW264.7 cells were transfected by LV-*gra15*_II_-Mφ or LV-Mφ as mock control. The results indicated that the Lv-mediated gene transfer system could efficiently transfer GRA15_II_ gene into RAW264.7 cells. As shown in Figure 1A, we observed numerous green fluorescent cells using the fluorescence microscope. Also, we detected the transfection efficiency using flow cytometry. The efficiency of LV-*gra15*_II_-Mφ, LV-Mφ, and Mφ was 90.02, 97.1, and 0.00%, respectively.

### ToxoGRA15_II_ Drove RAW264.7 Cells to M1

**Figure 2:**
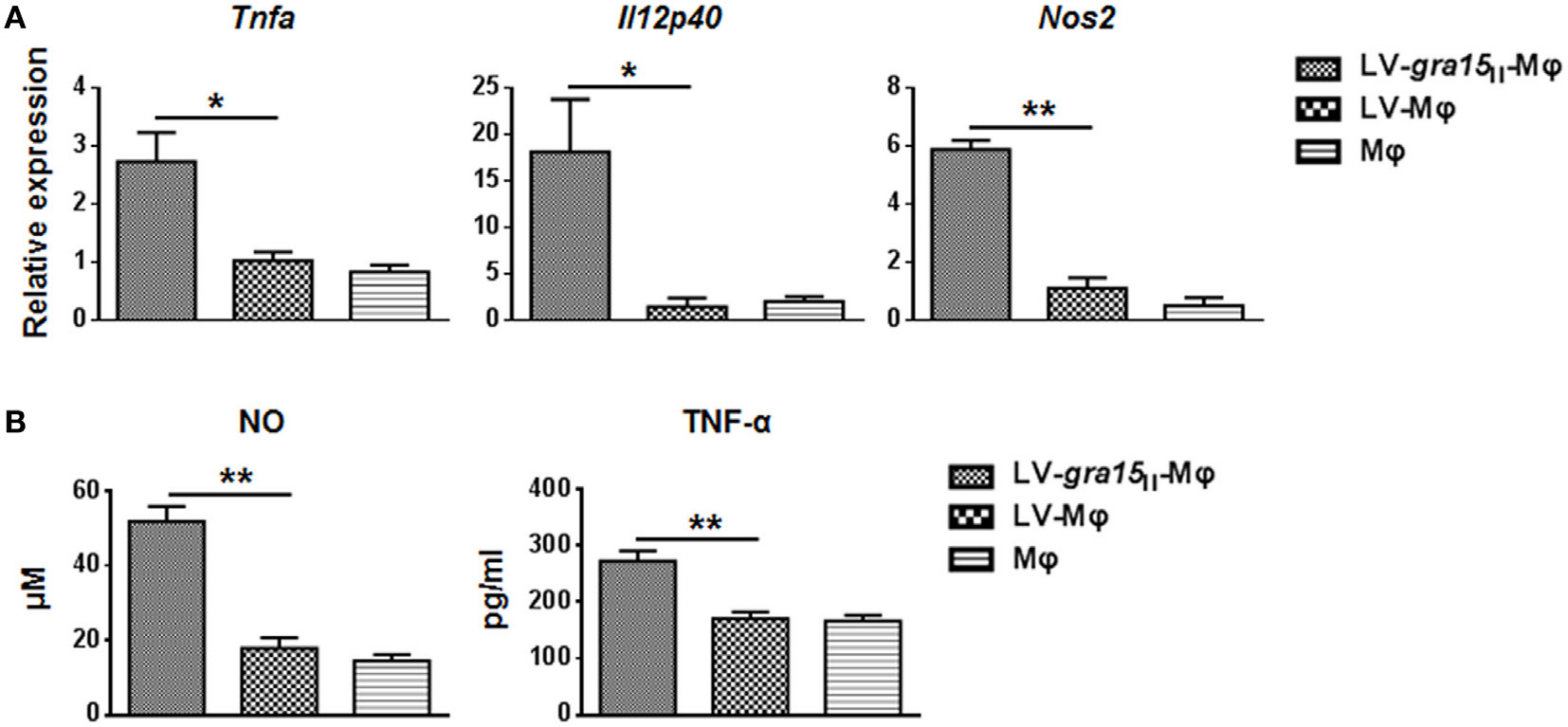
**Analysis of the iconic cytokines of the classically activated macrophages**. **(A)** The mRNA expression of LV-*gra15*_II_-Mφ upregulated TNF-α (*P* < 0.05), IL-12 (*P* < 0.05), and inducible nitric oxide synthase (iNOS) (*P* < 0.001), respectively, compared with LV-Mφ. **(B)** The concentration of nitric oxide (NO) and TNF-α in cell supernatants was tested with enzyme-linked immunosorbent assay (*P < 0.05, **P < 0.01).

Compared with LV-Mφ, LV-*gra15*_II_-Mφ expressed high level of PD-L1 (*P* < *0.01*) and CD80 (*P* < 0.01) determined by FCM (Figures 1B,C). The relative TNF-α (*P* < 0.05), IL-12 (*P* < 0.05), and iNOS (*P* < 0.001) mRNA expression levels in LV-*gra15*_II_-Mφ were significantly increased compared with LV-Mφ (Figure 2A). As for the cell supernatants, the inflammatory factors NO (*P* < 0.001) and TNF-α (*P* < 0.01) sharply rose in macrophages infected with LV-*gra15*_II_, which is in accordance with the mRNA expression (Figure 2B).

### LV-*gra15*_II_-Mφ Alleviated *In Vivo* Proliferation of the Tumor Cells

**Figure 3:**
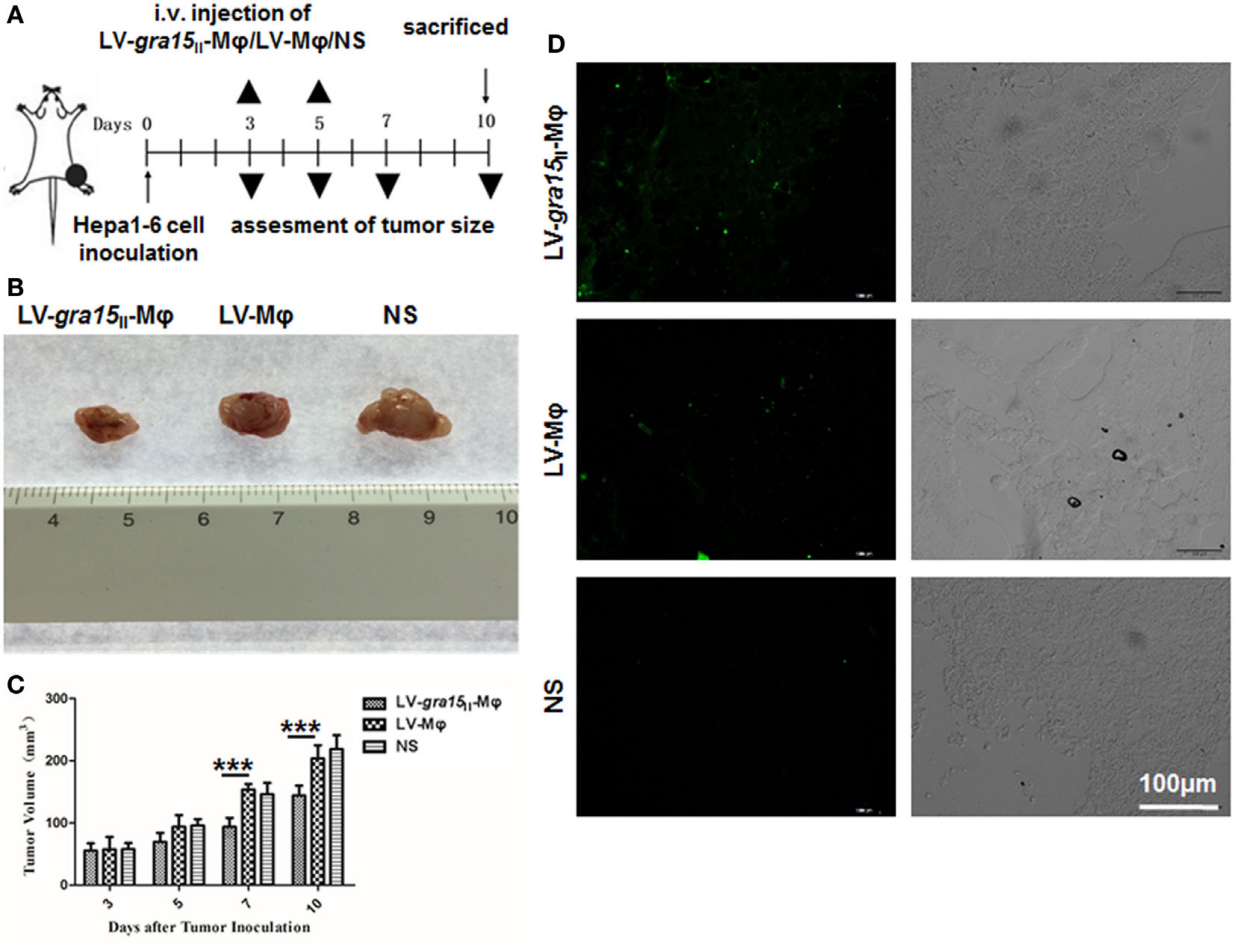
**LV-*gra15*_II_-Mφ inhibited tumor growth in the Hepa1-6 planted tumor model**. **(A)** Experimental outline for inhibitory tumorigenesis by administration of LV-*gra15*_II_-Mφ in tumor-bearing mice. LV-Mφ and saline were used as control. **(B)** Representative tumor in different groups. **(C)** Size of tumor. Data are representative of three independent experiments (***P < 0.001). **(D)** Tumor frozen sections were observed under the fluorescence microscope. Samples were collected 72 h after injection of LV-*gra15*_II_-Mφ, LV-Mφ, or normal saline (NS). The fluorescence microscopy fields shown are representative ones (10 fields per tumor, 3 tumors per group).

**Figure 4:**
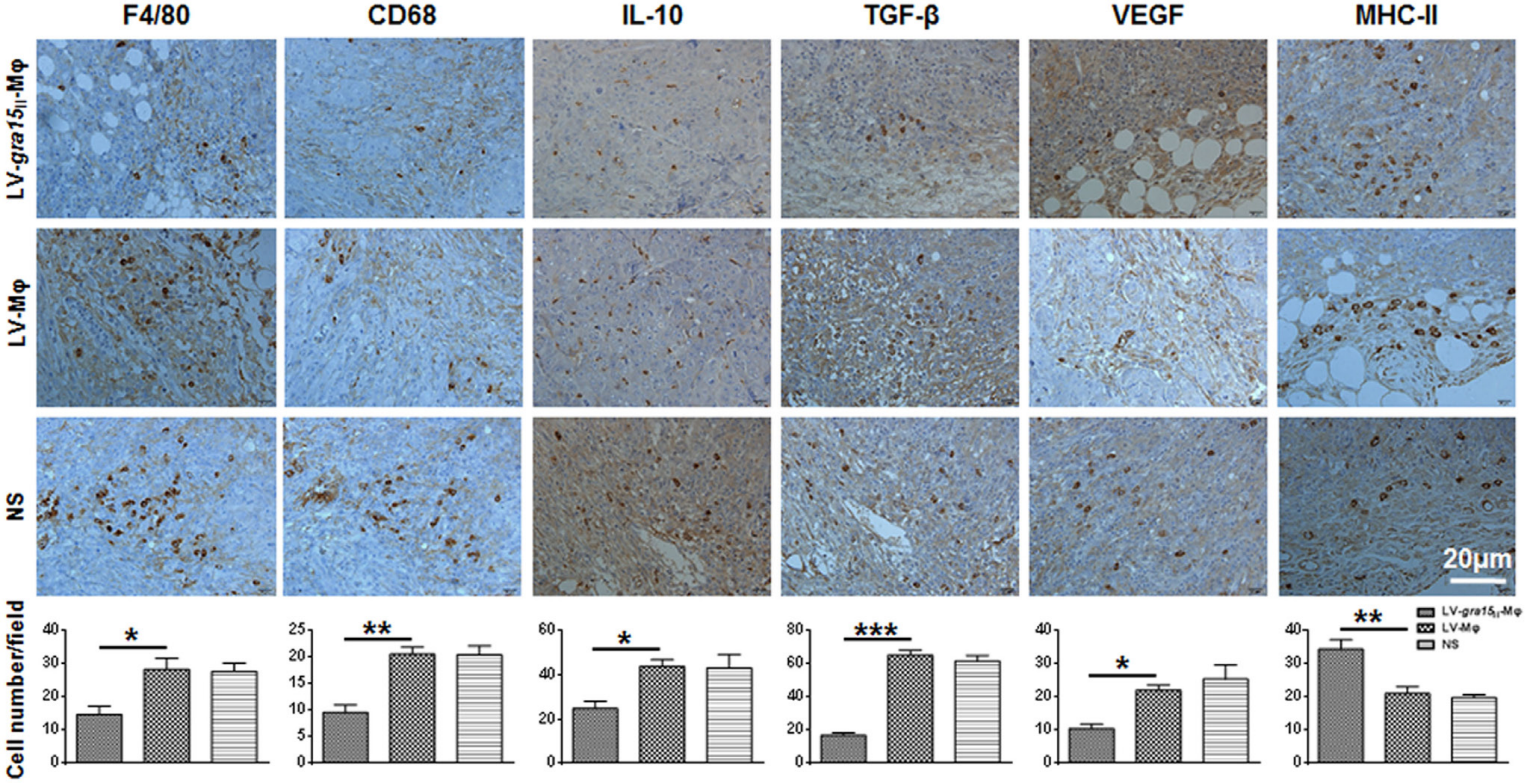
**Immunohistochemical analysis of tumor tissues after treatment with LV-*gra15*_II_-Mφ**. The macrophages were examined by immunostaining with the antibodies to F4/80 and CD68, and the number of these cells was counted at ×400 magnification. Data represent the mean Mφ number per field ± SD (10 fields per tumor, 3 tumors per group). The expression of interleukin-10 (IL-10), TGF-β, VEGF, and major histocompatibility complex II (MHCII) evaluated by immunostaining, and the number of IL-10-positive, TGF-β-positive, VEGF-positive, and MHCII-positive cells were counted (*P < 0.05, **P < 0.01, ***P < 0.001).

**Figure 5:**
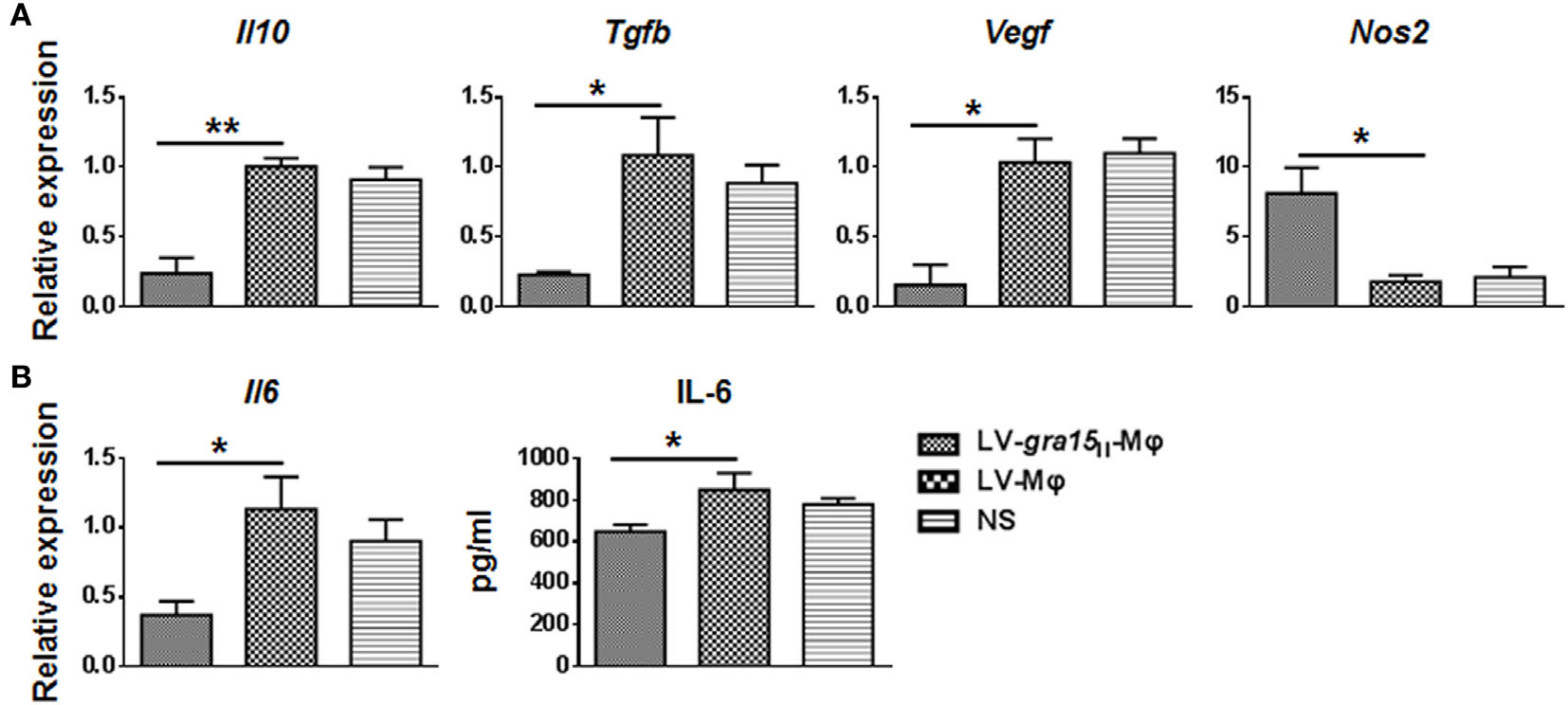
**Relative mRNA expression levels of immunosuppressive molecules and cytokines in tumor samples were assayed**. **(A)** The relative mRNA expressions of interleukin-10 (IL-10) (*P* < 0.01), TGF-β (*P* < 0.05), and VEGF (*P* < 0.05) were significantly decreased, and inducible nitric oxide synthase (iNOS) (*P* < 0.05) was remarkably increased in mice that received LV-*gra15*_II_-Mφ. **(B)** Interleukin-6 (IL-6) expression in both mRNA and protein was decreased in group LV-*gra15*_II_-Mφ.

Tumor was palpable, and tumor size was about 50 mm^3^ 3 days after Hepa1-6 cells subcutaneous injection in the mice (Figure 3A). Compared with the control group (LV-Mφ), on days 7 (*P* < 0.001) and 10 (*P* < 0.001) post Hepa1-6 inoculation, the tumor proliferation was markedly inhibited in mice that received twice LV-*gra15*_II_-Mφ treatments (Figures 3B,C). This result was obtained by measuring the size of the tumor (Figure 3C). Examination of frozen sections by fluorescence microscopy revealed that both LV-*gra15*_II_-Mφ and LV-Mφ reached the tumor site and remained in it for at least 72 h after intravenous injection (Figure 3D). The TAMs infiltration of solid tumor was examined by immunohistochemistry. Figure 4 showed that the number of macrophages in tumor microenvironment was decreased in the LV-*gra15*_II_-Mφ treated group, and reduced expression of IL-10, TGF-β, and VEGF in tumor tissues and increased expression of MHCII molecule in the animals that received LV-*gra15*_II_-Mφ inoculations, compared with the group treated with LV-Mφ. The data were further verified by using qRT-PCR. Consistent with the immunohistochemistry assay, relative mRNA expressions of IL-10 (*P* < 0.01), TGF-β (*P* < 0.05), and VEGF (*P* < 0.05) were significantly decreased, and iNOS (*P* < 0.05) was remarkably elevated (Figure 5A). More significantly, IL-6 production, which has been considered to be closely associated with tumorigenesis in tumor microenvironment, was impaired. The mice with LV-*gra15*_II_-Mφ injection exhibited a notable decrease in levels of mRNA coding for IL-6 (*P* < 0.05) when compared with the control group (Figure 5B). Parallel IL-6 expression results (*P* < 0.05) were confirmed by ELISA in the tumor homogenate supernatants (Figure 5B).

### LV-*gra15*_II_-Mφ Have Effects in Spleen

**Figure 6:**
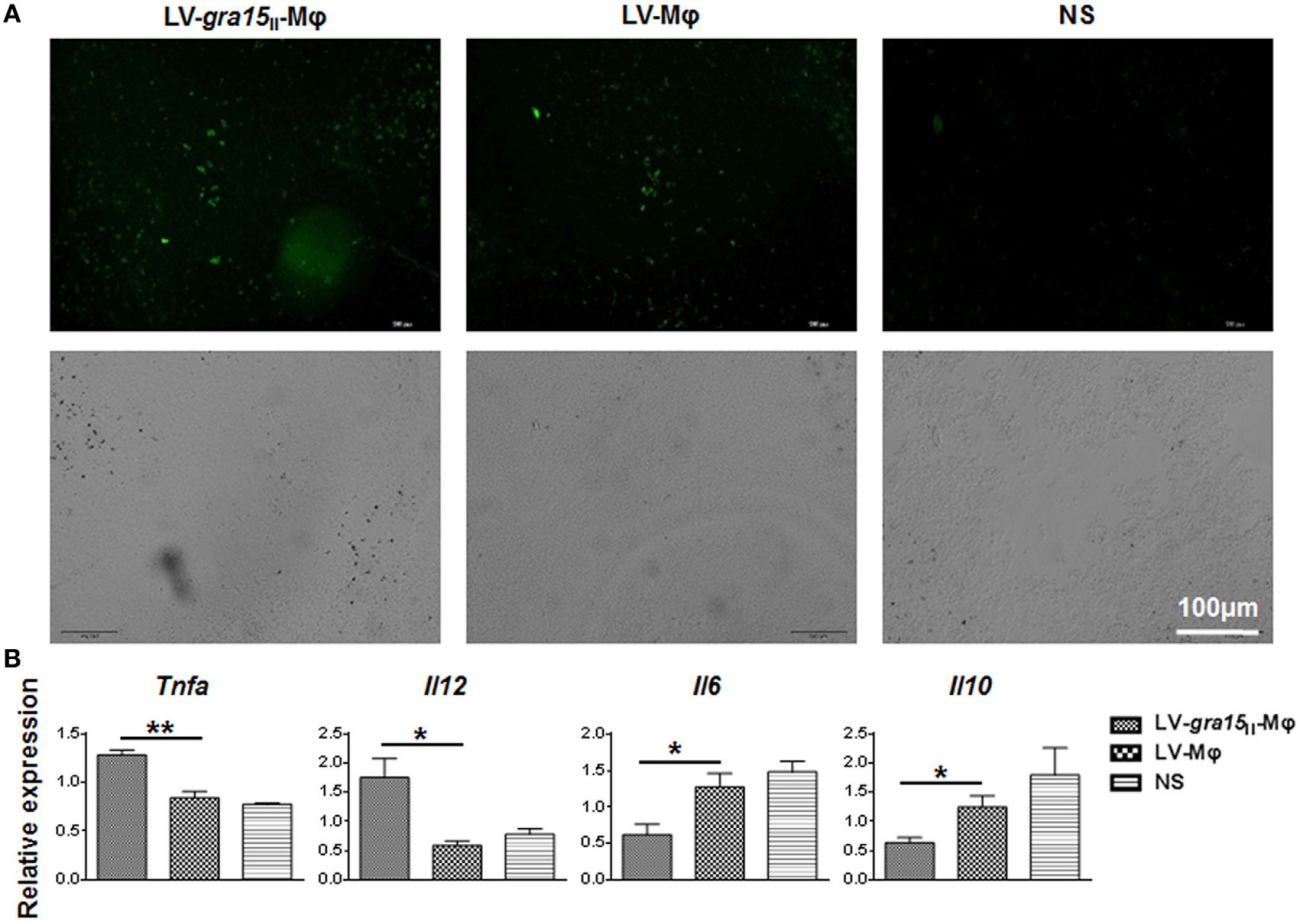
**LV-*gra15*_II_-Mφ reached spleen tissues, and relative mRNA expression levels of cytokines in spleen samples were assayed**. **(A)** Spleen frozen section were observed under the fluorescence microscope. Samples were collected 24 h after injection of LV-*gra15*_II_-Mφ, LV-Mφ, or normal saline (NS). The fluorescence microscopy fields shown are representative ones (10 fields per tumor, 3 tumors per group). **(B)** The relative mRNA expressions of TNF-α (*P* < 0.01) and IL-12 (*P* < 0.05) were significantly increased, interleukin-6 (IL-6) (*P* < 0.05) and interleukin-10 (IL-10) (*P* < 0.05) were remarkably decreased in mice that received LV-*gra15*_II_-Mφ.

Observation of frozen sections of spleen tissue under the fluorescence microscope revealed that both LV-*gra15*_II_-Mφ and LV-Mφ reached spleen and survived in this site after intravenous injection (Figure 6A). Mice injected with LV-*gra15*_II_-Mφ exhibited notable increase in mRNA transcription of TNF-α (*P* < 0.01) and IL-12 (*P* < 0.05), and decrease in IL-6 (*P* < 0.01) and IL-10 (*P* < 0.01), in comparison to mice injected with LV-Mφ (Figure 6B).

### LV-*gra15*_II_-Mφ Weakened the Migration and Invasion of Hepa1-6 *In Vitro*

**Figure 7:**
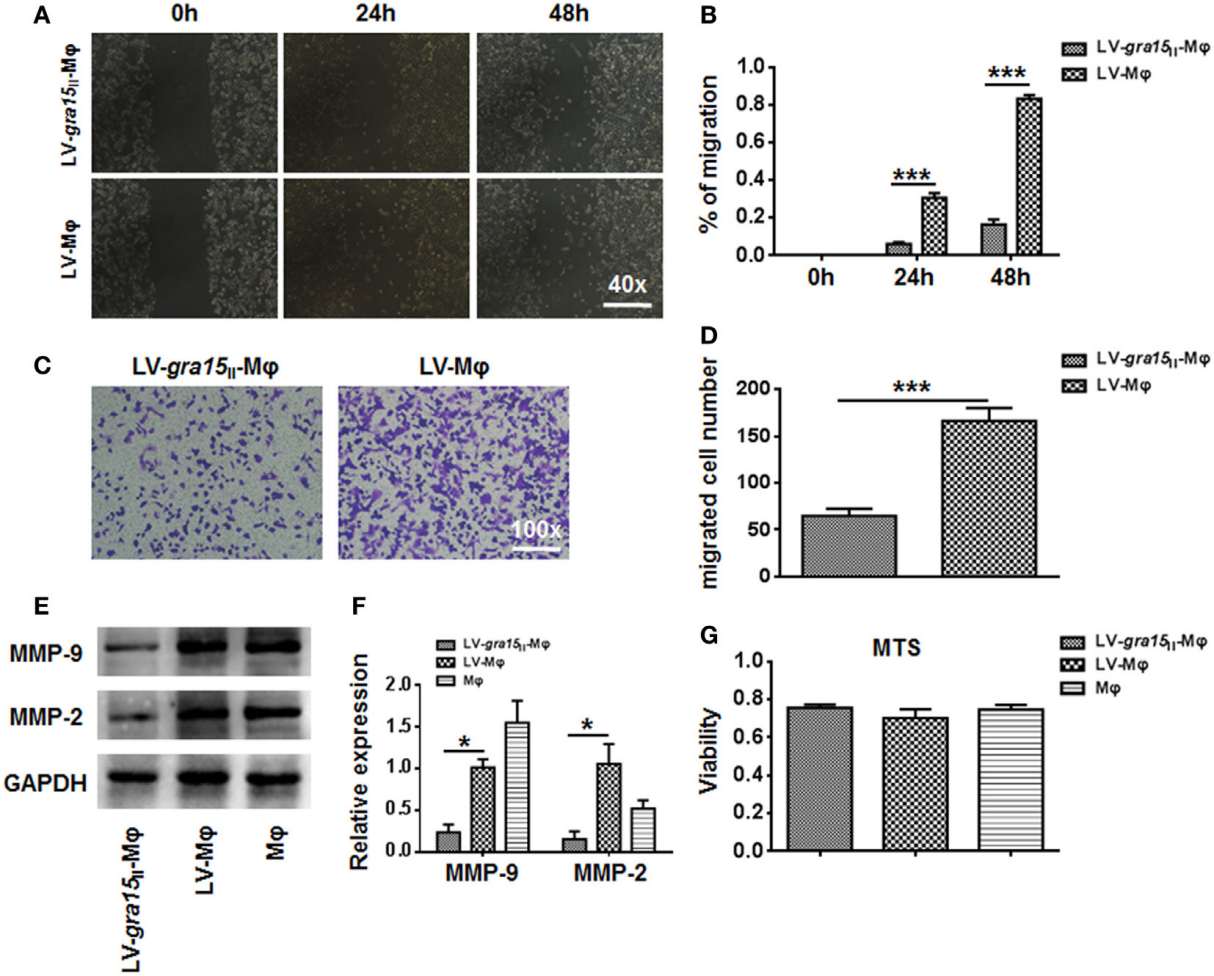
**The migration and invasion of Hepa1-6 cells were weakened after coculturing with LV-*gra15*_II_-Mφ**. **(A,B)** The migration of Hepa1-6 cells in the wound healing assay taken 24 and 48 h after coculture of Hepa1-6 with Mφ. **(C)** The representative images of Hepa1-6 cells migration after coculturing with LV-*gra15*_II_-Mφ (×100). **(D)** The number of migrated Hepa1-6 cells. **(E)** Western blotting analysis of matrix metalloproteinase (MMP)-9 and MMP-2 expression. **(F)** The mRNA expression of MMP-9 and MMP-2 in Hepa1-6 cells. **(G)** MTS assay in LV-*gra15*_II_-Mφ and LV-Mφ groups (*P < 0.05, ***P < 0.001).

Cocultured migration and invasion of Hepa1-6 cells with LV-*gra15*_II_-Mφ were inhibited. Obviously, in the wound healing assay, the relative Hepa1-6 cell migration was notably decreased in the LV-*gra15*_II_-Mφ coculture group (*P* < 0.001) compared with the LV-Mφ coculture group (Figures 7A,B). Similar results were obtained in the invasion assay, the migrated numbers of Hepa1-6 cells were declined in the group cocultured with LV-*gra15*_II_-Mφ (*P* < 0.001) (Figures 7C,D). The MMP-9 (*P* < 0.05) and MMP-2 (*P* < 0.05) mRNA expression levels in the Hepa1-6 cells were downregulated in the LV-*gra15*_II_-Mφ coculture group (Figure 7E). To verify the mRNA data, we identified the MMP-9 and MMP-2 expression by Western blotting. The results revealed that the levels of MMP-9 and MMP-2 were lowered in LV-*gra15*_II_-Mφ coculture group (Figure 7F). However, there was no noticeable apoptosis of Hepa1-6 cells that were cocultured with LV-*gra15*_II_-Mφ (Figure 7G).

## Discussion

Macrophages and related mononuclear phagocytes are the preferred host cells of *T. gondii in vivo*, although the intracellular parasite can infect any sort of nucleated cells. Macrophage-centered innate immune response of host to *T. gondii* may determine the outcomes of its infection. One of the recent key findings revealed that type II strains of *Toxoplasma* evoked a gene expression profile quite close to classically activated macrophages (M1) that induces strong IL-12- and Th1-predominant immunity crucial to the consequences of chronic infection (23). Previous investigators found that *T. gondii*-derived genotype-associated effector of ToxoGRA15_II_ is responsible for efficient induction of M1 phenotype (18), which is featured by presenting high NO, TNF-α, iNOS, and IL-12p40 (5, 8, 19–21, 24), involving in host innate immunity and Th1-dominant inflammatory response to the pathogen. Our previous study showed that ToxoGRA15_II_ is able to induce RAW264.7 polarization to M1, leading to distinct attenuation of liver fibrotic process after M1 transferring to mice infected with *Schistosoma japonicum* (21).

Chronic inflammation is known to contribute to cancer progression (25, 26). TAMs, recruited by chemokines CCL2, CCL5, and CXCL, are derived from circulating monocyte precursors (27) and are the most important regulators of tumorigenesis (28, 29), which may serve as one of the hallmarks of cancer. It has been well documented that TAMs promote the development of tumor, and its infiltration is highly correlated with poor prognosis (7, 30, 31). Here, we reported an apicomplexan parasite-derived peptide ToxoGRA15_II_, which may alter tumor microenvironment to inhibit tumor growth transplanted with Hepa1-6. In the present study, LV-*gra15_II_*-Mφ was injected through veins alleviated proliferation of the tumor in mice. Thereby, we found that M1 phenotype evoked by ToxoGRA15_II_ successfully reduced the volume of tumors and attenuated the infiltration of TAMs in tumor tissues of murine model. The number of macrophages labeled by F4/80 and CD68 in the group treated with LV-*gra15_II_*-Mφ was significantly lower than those in the group that received LV-Mφ. For instance, once M1 phenotype successfully activated in tumor microenvironment may play a significant role in restriction of tumor progression (27). The number of macrophages expressing MHCII increased in the animals with LV-*gra15_II_*-Mφ administration when compared to those with LV-Mφ, indicating an improved antigen-presenting capability. It has been well confirmed that M1 produce high levels of pro-inflammatory cytokines of IL-12 and NO, and characterized by MHCII molecules and functions as a key population of antitumor cells (32, 33).

RAW264.7 macrophage used here for transfusion is a common cell line of mouse macrophages for the study of immune responses to microbes and their products. However, we did not observe any remarkable allogeneic response of recipient C57BL/6 mice to the transferred cells. A possible explanation is that cancers are characterized by a compromised immunogenicity due to their genetic instability and epigenetically downregulated MHC receptor (34, 35). RAW264.7, as a cancer cell line after a long-term *in vitro* passage, should have a feature of low antigenicity and fail to induce strong allogeneic response of recipient C57BL/6 mice that had been concomitantly immunocompromised by tumor implantation. In the present study, neither C57BL/6 mice inoculated with LV-gra15_II_-Mφ nor with LV-Mφ mock macrophages presented distinct difference of general body condition compared to those of normal saline control, suggesting the tolerance of recipient’s C57 BL/6 mice to transferred cells, and the tumor growth restriction was not caused by allogeneic response of transferred macrophages. Additionally, we used MHCII monoclonal antibodies to recognize MHCII alleles expressed by transferred cells and the recipient’s cells (C57 BL/6 peritoneal exudate macrophages), respectively. The result showed that both cells were strongly recognized by the MHCII monoclonal antibodies used (data not shown). Taken together, we believe that passive transferring of RAW264.7 would not be able to evoke allogeneic response that is strong enough to injure the recipient animals.

The TAMs, which are mostly similar to alternatively activated macrophages (M2) in phenotype, are reeducated by tumor-derived factors and usually act as pro-tumor growth cells (36, 37). Cytokine and chemokine profiles in tumor microenvironment control the bias of infiltrating macrophages. To explore the putative retraining of the TAMs in tumor tissues, we detected the expressions of immune factors and those associated with tumor angiogenesis, such as IL-10, TGF-β, and VEGF. Furthermore, our results showed that mice that were treated with LV-*gra15*_II_-Mφ presented an obvious reduction of the molecules that are related to the downregulation of immune response. Previous literatures indicated that IL-6 is involved in facilitation of tumor growth *via* regulating all hallmarks of cancer and multiple signaling pathways (38, 39). Here, we noted a particular decrease of IL-6 production, which might also contribute to alteration of tumor microenvironment by M1 transferring.

In the present investigation, we tracked the *in vivo* fate of the LV-*gra15_II_*-Mφ and found the macrophages inoculated in both tumor and spleen under fluorescence microscope. We subsequently amplified GRA15_II_ mRNAs in tumor tissues and splenocytes with RT-PCR. Accordingly, both tumor and spleen isolated from the mice injected with LV-*gra15_II_*-Mφ present positive expression of GRA15_II_ (data not shown). It has been generally suggested that recruitment and infiltration of immune cells, including macrophages, dendritic cells (DCs), and T cells, to/in tumor tissues are involved in restricting tumor growth. We found that MHCII expression, a phenotype of macrophages and non-macrophages such as DCs and B cells, was apparently increased. We also noted remarkably elevated iNOS and NO production and decreased tumor growth-associated cytokines such as IL-10, TGF-β, IL-6, and VEGF in tumor tissues of recipient animals. All of these findings suggested that transferred M1 may be directly and indirectly involved in the inhibition effect on tumor development. Thus, we hypothesize that M1 driven by ToxoGRA15_II_ transfection may alter the TAM-dominated tumor microenvironment *via* effects on systemic immunity and possibly also *via* local effects at the tumor site.

It is known that PD-L1 and programmed death ligand 2 (PD-L2) are the ligands for programmed death 1 (40, 41). PD-L1 and PD-L2 are negative regulators, and both of them are involved in T cell activation (42) and cell-mediated immune response (43). Moreover, PD-L1 presents up-expression on inflammatory macrophages when exposed to LPS and IFN-γ (24). It was reported that CD80, the ligand on antigen-presenting cells, such as macrophages and DCs, is necessary for the amplification of inflammatory response (44). In the present study, we further detected that both co-stimulatory molecules CD80 and co-inhibitory molecule PD-L1 were highly expressed on LV-*gra15_II_*-Mφ compared with LV-Mφ and Mφ. However, either stimulatory CD80 or inhibitory PD-L1, which is responsible for direct induction of macrophage polarization, remains unclarified. The balance between these two opposite molecules, which might result in the macrophages skewing, still needs further investigation. Hypothetically, the higher expression of PD-l is a negative feedback regulation mechanism to defense an immune overreaction.

Matrix metalloproteinases belong to the multigene family of zinc-dependent endopeptidases of over 25 enzymes (45, 46). It has been demonstrated that MMPs are involved in stages of tumor invasion and metastasis (47, 48), suggesting a close relationship with immune cells (49). In the transwell assays, the secretion of MMP-2 and MMP-9 by the Hepa1-6 cells was dampened. Additionally, the competence of migration and invasion of Hepa1-6 cells was seemly inhibited after coculturing with LV-*gra15_II_*-Mφ. Further *in vivo* approaches are needed to determine the restrained invasion of Hepa1-6, which is in association with inhibition of MMPs expression of TAMs in solid tumors (27).

We conclude that ToxoGRA15_II_, as a strong inducer of classically activated macrophages and an applicable peptide derived from *T. gondii*, may have a potential role in increasing early antitumor immunity due to its ability to indigenously activate NF-κB signaling in macrophages and synchronically induce both highly potent innate as well as adaptive immune mechanisms for cancer therapy. A recent study revealed that *T. gondii* parasite with deletion of GRA15 did not lose its antitumor activity (50), indicating that other effectors might also be involved in suppression of tumor development. Further approaches are needed to confirm residing of the transferred macrophages, T cells, and DCs as well, in tumor tissues and their ability to reeducate TAMs and reset antitumor immunity in tumortherapy. Optimization study of GRA15_II_ delivery system is ongoing.

## Author Contributions

JS, YC, and YL conceived and designed the experiments and critically revised the manuscript. YL, FP, and JC performed the trials. All authors contributed to discussion of the results followed by writing and reviewing the manuscript.

## Conflict of Interest Statement

The authors declare that the research was conducted in the absence of any commercial or financial relationships that could be construed as a potential conflict of interest.
